# The Future of the Metropolitan Hospital Sunday Fund

**Published:** 1911-03-25

**Authors:** 


### The Hospital

A Journal of

The Medical Sciences and Hospital Administration.

New Series. No. 216, Vol. VIII. [No. 1288, Vol. XLIX.] SATURDAY, MARCH 25, 1911

### THE FUTURE OF THE METROPOLITAN HOSPITAL SUNDAY FUND.

On Tuesday, the 28th instant, the Council of this Fund will meet to determine its attitude towards the hospitals which have organised Sunday performances at the cinematograph exhibitions as a new source of revenue. Ninety-seven hospitals received grants from the Hospital Sunday Fund in 1910, and of this number five, namely, the Evelina Hospital, the Hospital for Epilepsy and Paralysis, the Kingsland Hospital, the London Hospital, and the Poplar Hospital have so far exploited this source of income. It is admitted that the largest sum yet received by a hospital from this source in one year amounts to some 1,400*l*. The question, so far as the hospitals are concerned, is largely a financial one, and seeing that only nine hospitals out of ninety-seven received from this Fund a larger grant last year than 1,400*l*., it is presumable that the ninety-two hospitals who have refrained from exploiting this source of income have probably good reasons for their inaction in the matter.

The regulations of the County Council forbid the opening of cinematograph exhibitions on Sunday for the benefit of a hospital which the Council may approve, except a request is received from the hospital authorities to permit such exhibitions to be held on Sunday in its support. These facts make it clear that the present danger to the successful maintenance of the Hospital Sunday Fund collections in places of worship, whatever it may amount to, has been caused by the action of the managers of those hospitals which at present benefit by these exhibitions. Law IX. of the Laws of the Constitution of the Hospital Sunday Fund provides that any collection made in a place of worship on Hospital Sunday for a particular hospital or institution participating in the Hospital Sunday Fund, and not sent to the treasurer of this Fund, shall be deducted from the grant made by it to such a hospital. Providing it can be proved that the opening of cinematograph exhibitions on Sunday in support of hospitals is so strongly objected to by a large and increasing number of congregations at present associated with Hospital Sunday that they will withdraw or reduce or withhold their contributions in future, precedent would warrant an amendment of Law IX. to meet this new danger to the continuance and prosperity of Hospital Sunday in London. The position, therefore, is that by the deliberate action of certain hospitals the success of the Hospital Sunday Fund, and even its continuance, may largely depend upon the action which the Council of the Fund decides to take at Tuesday's meeting.

We hope that the Council will pass no resolution which commits it to an interference, however indirect or remote, (1) with the free action of the voluntary hospitals in raising funds, or (2) with the free action of the contributing congregations in sending conditional grants to the Fund, or (3) to an expression of opinion as to the policy of encouraging or discouraging cinematograph exhibitions on Sunday. The Hospital Sunday Fund can have and ought to have no opinion upon matters of this kind, which are outside their province as trustees, who receive free gifts from the churches of all denominations and distribute them to the great medical institutions throughout the Metropolis. The Hospital Sunday Fund was founded to maintain a neutral platform where the representatives of all the churches can meet and unite in providing for the relief of the sick and suffering, who need such assistance. We regret to see the discussion so far, perhaps not unnaturally, has tended to include many points which are not, and can never be, properly dealt with by the Hospital Sunday Fund Council, for they form no part of its business. We hope, therefore, that time may not be wasted upon them at the meeting on the 28th instant.

The Sunday Fund Council are in a difficult position because they may place the Fund, if great prudence and caution are not exercised, in the very centre of a controversy with which it properly has nothing whatever to do. On the one hand there are the five Hospitals, whose action has brought about the present crisis. The managers of these Hospitals are perfectly justified in using every legitimate means to increase their income, but they must at the same time take the whole responsibility for their action and its consequences. If the effect of the course they have adopted should prove, in practice, seriously to threaten the permanence of the Hospital Sunday Fund in London, it is to be hoped that, on public grounds and as a matter of business, the gentlemen responsible will not consent to imperil an annual hospital revenue of £40,000 a year from congregational collections for the sake of gaining a sum which, in the aggregate, may not amount to more than a tenth of the total raised in the churches on Hospital Sunday.

Again the discussion of this question has brought to light two opposing camps. One, that of certain churches and their ministers who object altogether to the opening of cinematograph exhibitions on Sunday, and two, that large section of the general public, including many ministers of religion, who hold that such exhibitions are not only not harmful but that they may under proper management fulfil a useful and even a moral purpose. The Council of the Sunday Fund should avoid any expression of opinion in favour of one side or the other, for to do so, as trustees, would be *ultra vires* on their part. At the same time the Council must take care to protect the Fund as far as it may be possible from any risk of disaster. Such is the problem which the Hospital Sunday Fund Council will have to face on Tuesday. Its solution in our judgment lies in a plain declaration that the Council are not prepared to fetter the free action of the hospitals in raising income, nor to interfere with the free action of the churches in attaching conditions to any grant which they may send to the Mansion House for distribution by the Sunday Fund. It must be made plain that the Hospital Sunday Fund is not opposed to the provision of adequate and wholesome amusement for the people on Sundays, and that it declines to interfere with the sources of revenue a given hospital may provide. The course we suggest would remove any possibility of misapprehension, whilst it would cast the whole responsibility for any consequences upon those hospitals which alone are responsible for the creation of the present crisis.

